# The Influence of Socioeconomic Status on Esophageal Cancer in Taiwan: A Population-Based Study

**DOI:** 10.3390/jpm12040595

**Published:** 2022-04-07

**Authors:** Hao-Yun Chen, I-Chieh Chen, Yi-Huei Chen, Chi-Chang Chen, Cheng-Yen Chuang, Ching-Heng Lin

**Affiliations:** 1 Department of Thoracic Surgery, Taichung Veterans General Hospital, Taichung 40705, Taiwan; bcdhow@hotmail.com; 2 Department of Medical Research, Taichung Veterans General Hospital, Taichung 40705, Taiwan; icchen@vghtc.gov.tw (I.-C.C.); chenyh@vghtc.gov.tw (Y.-H.C.); 3 Department of Medical Education, Taichung Veterans General Hospital, Taichung 40705, Taiwan; clayton0805@gmail.com; 4 Department of Industrial Engineering and Enterprise Information, Tunghai University, Taichung 40704, Taiwan; 5 Department of Radiology, Taichung Veterans General Hospital, Taichung 40705, Taiwan; 6 Medical Imaging Department, China Medical University Hospital, Taichung 404332, Taiwan; 7 Department of Health Care Management, National Taipei University of Nursing and Health Sciences, Taipei 11219, Taiwan; 8 Department of Public Health, College of Medicine, Fu Jen Catholic University, New Taipei City 24205, Taiwan; 9 Institute of Public Health and Community Medicine Research Center, National Yang Ming Chiao Tung University, Taipei 11221, Taiwan; 10 Department of Medical Research, China Medical University Hospital, Taichung 40402, Taiwan

**Keywords:** socioeconomic status, esophageal cancer, esophageal squamous cell carcinoma, esophageal adenocarcinoma

## Abstract

Background: Esophageal cancer has extreme worldwide demographic and histologic variations in occurrence; thus, understanding the pathogenesis of esophageal cancer must be region- or country-based. We examined the incidence and tumor stage at diagnosis of esophageal cancer in relation to patients’ socioeconomic status (SES) in Taiwan. Method: This retrospective cohort study used data from Taiwan’s National Health Insurance Research Database and Taiwan Cancer Registry collected between January 2008 and December 2014. The records of 40- to 79-year-old patients diagnosed with esophageal cancer were retrieved. The distribution of the crude incidence rates of esophageal cancer by occupation and income variables was studied retrospectively. Cox proportional hazard model was used to adjust for potential confounders and compare the esophageal cancer incidence among four independent variables: age, gender, occupation, and income. Logistic regression analysis was applied to find the power of the independent variables on the odds ratio of late-stage presentation. Results: The analysis included 7763 subjects. Esophageal squamous cell carcinoma (ESCC) was the predominant histological type (96.6%) and 94.4% of patients were male. The peak affected age for ESCC was 50 to 59 years, whereas the risk of esophageal adenocarcinoma increased progressively with age. The risk of ESCC was significantly unfavorable for the most disadvantaged group, either in occupation or income, while in EAC, risk was unrelated to either factor. The stage of cancer at diagnosis was lower in the highest income groups than in the other two groups. Conclusion: Significant SES disparities in esophageal cancer incidence, based on occupation and income, are present in Taiwan. Low SES populations have a higher percentage of late-stage diagnosis. Resolution of the increasing socioeconomic disparities and narrowing the gaps in health inequities in Taiwan are needed.

## 1. Introduction

Esophageal cancer is one of the most prevalent types of cancer and is an increasing worldwide problem; it is the seventh most common cancer and the sixth leading cause of death from cancer [1,2], which reflects its life-threatening behavior. In 2018, there were an estimated 572,000 cases of esophageal cancer globally, resulting in more than 508,000 deaths [1]. The overall 5-year survival rate in patients amenable to definitive treatment is poor, varying from 5% to 30% [2]. Early diagnosis and complete resection are the only ways to combat esophageal cancer clinically, while prevention is the mainstay to avert it.

The rising incidence of esophageal cancer in recent decades has been accompanied by a changing dominance of histologic type. Two histologic types account for most malignant esophageal neoplasms: esophageal adenocarcinoma (EAC) and esophageal squamous cell carcinoma (ESCC) [3]. Worldwide, ESCC remains the dominant histology, but EAC is now more prevalent than ESCC in the United States and Western Europe [2,4,5]. The incidence of esophageal cancer in Eastern Asia, such as in Taiwan, is increased in the region called the Esophageal Cancer Belt in the world map, where ESCC is still the predominant type, and its prevalence will likely increase because of increased tobacco and alcohol consumption [6]. The cause for the demographic shift from dominant ESCC to EAC in parts of the world is unclear. The extreme geographical and histological variation in the incidence rates of esophageal cancer make understanding its pathophysiology and management difficult.

Socioeconomic status (SES) and health disparities are strongly correlated, and efficient and effective policies that reduce the impact of a disease require understanding the relationship between SES and disease [7,8,9]. Associations between SES and morbidity, such as in cancer, have been reported [10,11]. Income, education, and occupation are accepted measures of social class [10]. SES is a complicated construct [12,13] and the use of various measures of SES make comparisons and interpretations difficult.

Most cancers run an insidious and progressive course. Recent large-scale epidemiological studies reveal that their nature can be correlated with global lifestyle changes. Epidemiological studies are important in portraying a broad view of disease, but the complex effects of time and space [13,14] make successive studies in various localities and epochs necessary. Studies of this subject have been conducted in Western countries [15,16,17] and in Asian populations [18,19], but few studies have investigated the association between SES and esophageal cancer in Taiwan [20,21].

In view of differences between Taiwan and other developed countries, the increase in cases of esophageal cancer, the types of cancer, the clinical stage at diagnosis, and the association with SES and esophageal cancer deserve examination and clarification. The goal of this study was to characterize esophageal cancer and the association of incidence and tumor stage at diagnosis with SES in Taiwan. The results of this study may accelerate the development of more robust policies to reduce the occurrence of esophageal cancer and the delivery of better management of the disease among the less healthy population, as well as provide comparisons of esophageal cancer between Taiwan and other countries.

## 2. Materials and Methods

### 2.1. Ethical Approval

This study was approved by the Institutional Review Board of Taichung Veterans General Hospital (IRB No. CE17201A). All the data were anonymised, and informed consent was hence waived.

### 2.2. Patient and Public Involvement

This research was performed without patient involvement. Patients were not involved with regards to the design of the study, measurement of the outcome, or interpretation of the results.

### 2.3. Data Source

All data were obtained from the National Health Insurance Research Database (NHIRD) and the Taiwan Cancer Registry (TCR). The NHIRD was implemented in March 1995 and provides mandatory universal health insurance for over 23 million citizens in Taiwan, representing 99% of the total population [22]. The plan has contracts with 97% of medical providers. The National Health Insurance Bureau of Taiwan randomly checked the charts, 1 for every 100 outpatients and 1 for every 20 inpatients, and interviewed the patients to verify the accuracy of the diagnosis. All beneficiaries can be classified into 15 social statuses when joining the NHIRD, based on their lifestyle, specific identity, or occupation. Among them, government employees, enterprise employees and labor members constitute the major categories. The NHIRD is a large representative health care database, and its validity and clinical consistency in cancer research have been proven [23]. The TCR was established in 1979 by the Ministry of Health and Welfare. Mandatory reporting of specific newly diagnosed cancers in hospitals that can accommodate more than 50 beds is required by TCR [24]. These two databases can be cross-linked by a unique encryption identity number under the approval of the Department of Statistics, Ministry of Health and Welfare of Taiwan. As the source data used in this study were totally de-identified and encrypted to protect patient privacy, the requirement for obtaining patient consent was waived.

### 2.4. Study Design

**Figure 1:**
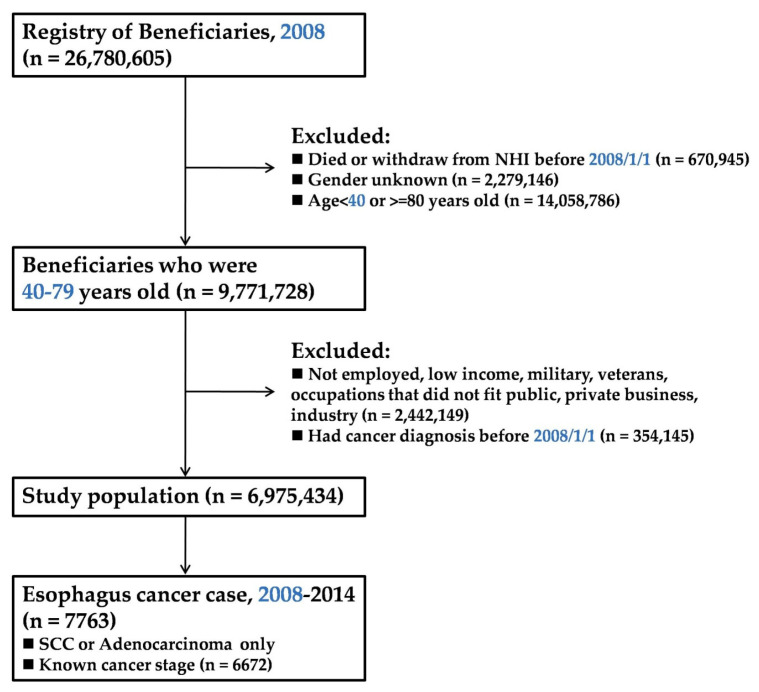
Flow chart for selection of the study population.

Subjects with social status belonging to the categories of government employee, enterprise employee, or labor class (Figure 1) were retrieved, and the other 12 status categories were not included due to the large difference in special characteristics and income structure of each category. All the included subjects were salaried employees with minimum wage of 17,280 Taiwan dollars (TWD) per month, or approximately 580 USD. Subjects who were diagnosed with esophageal cancer from 1 January 2008 to 31 December 2014, based on the International Classification of Diseases, Ninth Revision, Clinical Modification (ICD-9-CM) codes 150.01–150.09 were included in this study; subjects who had pre-existing cancer diagnoses were excluded. In addition, esophageal cancer cases with complete staging, classified as early stage (stage I and II) and late stage (stage III and IV) groups, were collected from the TCR database.

### 2.5. Socioeconomic Stratifications

Four independent variables were investigated: age, gender, occupation, and income. The latter two variables were regarded as common measures for stratifying SES. The occupational variable had three classes: (1) Government employees, mainly composed of civil servants and teaching staff with undergraduate degrees or above, who may have the most security in their jobs and be better paid; (2) members of the labor classes, consisting of farmers, fishermen, and so-called blue-collar workers, who may be less educated, earn less, and have less job security; (3) enterprise employees who have intermediate-level education, income, and job security. The employees’ monthly wages were reflected in their insurance premiums, which were based on their reported wage. In the NHIRD, salary data are divided into 54 levels at 10 different intervals. To minimize data manipulation, we used the quantile of the total subject salary data to divide the study population into high-, medium-, and low-income levels. Because the income distribution is positively skewed, meaning that the objects covered are more concentrated in the low-income group, significantly more people were in the low-income group than in the other two groups. To have reasonable case distribution in each group for comparison, we set cutoff points for monthly wages of 31,800 TWD and 21,000 TWD to make the number of patients in each group a ratio of approximately 1:1:2 from high to low income, respectively (Table 1). The patients included in this study received a monthly income of at least 17,281TWD. The outpatient cost for esophageal patients was between 230 and 570 TWD. We defined high income as monthly wages exceeding 31,800 TWD, middle income as between 21,001 and 31,800 TWD, and low income as between 17,281 TWD (the minimum wage) and 21,000 TWD.

### 2.6. Statistical Analysis

We first used the chi-square test to demonstrate the characteristic compositions of the ESCC and EAC groups. We then conducted multivariable analyses to test the influence of age, gender, occupation, and income on the two histological types of esophageal cancer. The effects of the four variables on the incidence rate of esophageal cancer were illustrated by Cox proportional hazard model analysis. Additionally, we applied logistic regression analyses to determine the power of the independent variables on the odds ratio (OR) of late-stage presentation. ORs with 95% confidence intervals (CIs) were estimated.

The crude incidence rate of occupation and income group and the distribution of esophageal cancer cases were probed separately. The crude incidence rate of 100,000 person-years was estimated by dividing the number of incident esophageal cancer patients by the total person-years in the at-risk population during a specified year. The incident esophageal cancer cases were defined as patients with a record of esophageal cancer in a specified year but without a diagnosis of esophageal cancer prior to 1 January of that year. People with no history of esophageal cancer during the same year were defined as the at-risk population.

Statistical significance was set at a *p* value less than 0.05. SAS version 9.4 software (SAS Institute Inc., Cary, NC, USA) was used to perform all statistical analyses. We searched and processed all data in the Data Science Center of the Ministry of Health and Welfare, Taipei City 115204, Taiwan.

## 3. Results

As shown in Figure 1, the total eligible population consisted of 26,780,605 NHI-registered beneficiaries in 2008. Excluding those who withdrew from the NHI or died before 1 January that year and members with no gender record, 9,771,728 subjects were in the age range of 40 to 79 years, the range most affected by esophageal neoplasm. Following the occupational status inclusion criteria, 6,975,434 subjects (71.4%) met our criteria for analysis.

By linking the NHIRD and TCR databases, 7763 newly diagnosed ESCC (7500 cases, 96.6%) and EAC (263 cases, 3.4%) were used to determine the incidence rate. Of these, 6672 cases (approximately 86%) had complete staging information and were qualified in the OR analysis for late-stage presentation. Table 1 lists the basic characteristics of subjects with ESCC or EAC according to age, gender, occupation, and income. The highest proportion of esophageal cancer patients were males, aged 50–59 years, laborers, and low-income earners (17,281–21,000 TWD).

### 3.1. Esophageal Cancer Diagnoses of the 40- to 79-Year-Old Population in Taiwan

The crude incidence rate of esophageal cancer in 40- to 79-year-old people was 8.16/100,000 person-years (PYs), with 15.77/100,000 person-years (PYs) in ESCC and 0.55/100,000 person-years (PYs) in EAC (Table 2).

The highest crude esophageal cancer incidence rates in both ESCC and EAC were found in labor and low-income groups. According to distribution of stage, 77.1% of esophageal cancer cases were in the late stage at diagnosis (stage 3 or 4) (Table 3). Additionally, late-stage presentation was more common in patients with ESCC than in those with EAC.

We used a multivariable Cox proportional hazard model to evaluate the impact of variable factors on the incidence of esophageal cancer. As illustrated in Table 4, the incidence of ESCC was significantly increased in 50- to 69-year-old people (HR:1.47, 95% CI = 1.39–1.55, *p*  <  0.001, in age 50–59; HR:1.44, 95% CI = 1.35–1.54, *p* < 0.001 in age 60–69), while the incidence of EAC significantly increased progressively with age (HR:2.18 in age 50–59, 3.50 in age 60–69; and 5.69 in age 70–79, *p*  <  0.001), as verified with the Cochran-Armitage test (*p* < 0.001). Male subjects had significantly more both ESCC and EAC than female subjects, with hazard ratio of 19.25 (95% CI = 17.39–21.3, *p*  <  0.001) for ESCC and 6.37 (95% CI = 4.5–9.03, *p*  <  0.001)for EAC.

### 3.2. SES and Esophageal Cancer Diagnoses

**Figure 2:**
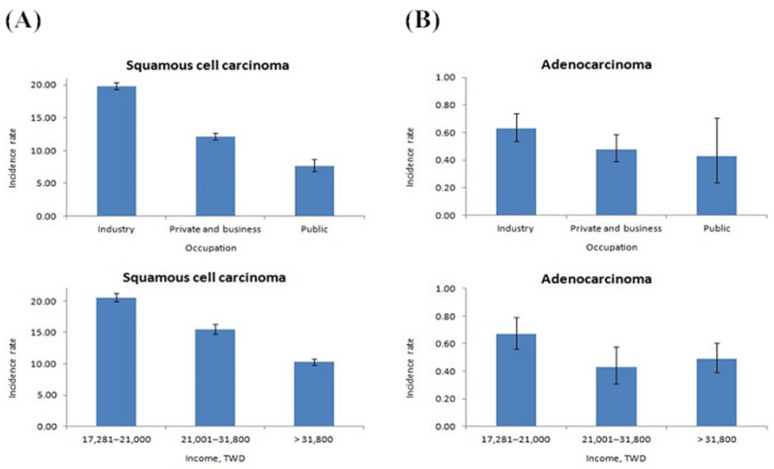
The crude incidence rate of occupation and income group in esophageal cancer. (**A**) The crude incidence rate of ESCC between occupation and income. (**B**) The crude incidence rate of EAC between occupation and income.

Occupational and wage-weighted SES were inversely associated with the incidence of ESCC, as shown in Figure 2A. The HR of either enterprise employees or government employees compared with laborers in ESCC was relatively low (HR: 0.76 and 0.58, *p*  <  0.001), and the incidence was negatively correlated with income level (HR: 0.91 in the middle-income group and 0.56 in the high-income group, *p*  <  0.001) (Table 4). However, neither occupation nor income was related to the incidence of EAC (Figure 2B).

### 3.3. Variables Affecting Late-Stage Esophageal Cancer at Diagnosis

We performed multivariable logistic regression to analyze the stage of esophageal cancer at diagnosis. As shown in Table 5, the risk of late-stage (Stage III + IV) diagnosis in ESCC was significantly higher in males (OR:1.88, 95% CI = 1.48–2.38) than in females. The risk of Stage III + IV EAC in males and females was not significantly different (OR: 0.47, 95% CI = 0.16–1.35). With respect to economic conditions, the highest income group, compared with the lowest income group (17,281–21,000 TWD), had the lowest odds ratio of late-stage diagnosis in ESCC (OR: 0.82, 95% CI = 0.68–0.98, *p* = 0.034), indicating that the low SES population had a higher percentage of late-stage diagnosis. There was also a tendency toward a lower incidence of late-stage EAC in the highest income population, but this difference did not reach statistical significance (OR: 0.39, 95% CI = 0.14–1.03, *p* = 0.057). Occupation was not correlated with the incidence of either ESCC or EAC.

## 4. Discussion

Throughout the world, the occurrence and outcomes of cancer vary considerably between racial and ethnic groups, largely because of inequalities in wealth that lead to differences in risk-factor exposures and barriers to high-quality cancer prevention, detection, and treatment. In this study, we identified unique features in the incidence and roles of SES in the presentation of esophageal cancer in Taiwan.

Esophageal cancer has exceptionally wide regional variability. According to the TCR, in 2012, the incidence rate of esophageal cancer in Taiwan was 13.8/100,000 PYs in males and 1.02/100,000 PYs in females, which, although not the world’s highest, is remarkable. To our knowledge, the extraordinarily high male:female ratio (13.6) exceeds any in the English literature [25]. With the rising incidence of esophageal cancer in recent decades, there has been a coincidental shift in the dominant histologic type. The prevalence of ESCC has been steadily declining in North America and Europe, probably because of decreased alcohol and tobacco use [26], whereas the incidence of EAC has been increasing rapidly in the United States, Australia, France, and England in recent decades [27,28]. In Asia, on the other hand, the rate of ESCC has increased constantly [28]. In Taiwan, according to cumulative data from the TCR, the incidence rate of esophageal cancer was 4.11/100,000 PYs in 1996 and 8.13/100,000PYs in 2007. ESCC was evidently responsible for this increase since the incidence of EAC remained unchanged [6]. Additionally, the main known risk factors for EAC are obesity, chronic gastroesophageal reflux disease (GERD) and Barrett’s esophagus. GERD can cause metaplastic changes to the esophagus, referred to as Barrett esophagus, that predispose patients to dysplasia and adenocarcinoma [29]. However, according to U.S. CDC, the prevalence of obesity in adults was 35.7% in 2009–2010. In contrast, the incidence of adult obesity in Taiwan reported by MOHW was 6% in the comparable period. Thus, the lower obesity rate could be the cause of the lower EAC incidence in Taiwan. Furthermore, genetic susceptibility may play a role in esophageal cancer development [30]. Immigrant epidemiological studies have supported the idea that genetic factors are important components affecting esophageal cancer risk.

In this study in Taiwan, the incidence of ESCC was highest in the 50 to 69 years old age range (HR:1.47 in aged 50–59 and 1.44 in aged 60–69), while the incidence of EAC was significantly increased with age (HR:2.18 in aged 50–59, 3.50 in aged 60–69, and 5.69 in aged 70–79, *p*  <  0.001). In other Asian countries, where ESCC is also predominant, the peak affected age of esophageal cancer is75 to 80 years in China and 60 to 70 years in Japan [31,32]. The explanation for this difference is lacking.

Worldwide, ESCC remains the predominant histological subtype, accounting for over 80% of esophageal cancer cases in recent decades. However, in the United States and Western Europe, EAC is now more prevalent than ESCC [27,28]. On the other hand, in Eastern Asia, a region called the Esophageal Cancer Belt, which includes portions of Iran, central Asia and North-Central China, ESCC is still the predominant type, contributing to 90% of cases [33]. Countries in Eastern and South-East Asia, as well as sub-Saharan Africa, have strikingly low EAC:ESCC incidence ratios (0.03–0.06), in contrast to countries in Northern America, Northern Europe and Western Europe that have EAC:ESCC incidence ratios of 0.3–2.8 [34]. It has been proposed that nations with higher socioeconomic development have higher EAC:ESCC incidence ratios [33], which does not pertain to Taiwan, a developed country, where our data reveal that ESCC is still predominant (EAC:ESCC incidence ratio = 0.035).

In Asia, the incidence of ESCC in males is higher than that in females [34]. However, in Linxian, China, and Golestan, Iran, the incidence of ESCC is similar in men and women. In these areas, the proportion of women who smoke cigarettes and drink alcohol is low [35], indicating that factors other than smoking and drinking affect the prevalence of ESCC in those regions and need to be clarified [36]. The global average incidence ratio for ESCC of males to females is 2.7:1, but there is a large difference from 1.2:1 in North Africa and West Asia to 7.8:1 in Eastern Europe [37]. The higher incidence of ESCC in men is at least partly due to differences in the rates of alcoholism and smoking between the sexes [38,39]. Surprisingly, in Taiwan, the male-to-female incidence ratio of 18:1 in favor of ESCC is extraordinarily high; we believe that the sex differences in esophageal cancer in Taiwan are due to exposure to alcohol, betel-nut and cigarette consumption.

A study conducted in Taiwan, analyzing 12,482 patients with esophageal cancer diagnosed between 1998 and 2007, found higher mortality rates in patients with lower income [20]. A study that enrolled 4097 Taiwanese esophageal cancer patients younger than 65 years old from 2002 to 2006 reported that higher individual socioeconomic status, defined by occupational category, was associated with a reduced five-year mortality rate [21]. Our study can be considered a consecutive, comprehensive, contemporary perspective of the association between the incidence and late-stage diagnosis of esophageal cancer with SES in Taiwan. The results show that the risk of ESCC, the major histological type of esophageal cancer in Taiwan (96.6%), is strongly correlated with occupation and income, and the odds ratio of late-stage diagnosis is also in favor of high-income persons. However, for EAC, only a tendency in favor of the high-income population in late-stage diagnosis was found. Since tumor stage at diagnosis is the strongest prognostic factor for esophageal cancer, the unfavorable survival rate of patients with low SES may be the consequence of delays in diagnosis and treatment [15]. Perhaps, by improving the SES of the low-SES population, the incidence of esophageal cancer and prognosis of the disease can be improved. It may not be possible to change some factors that affect SES in the short term, such as the public economy, to which funding of the public health plan is linked. However, if SES affects health outcomes through its influence on behavior and lifestyle, perhaps the incidence of ESCC can be reduced through educational programs that increase awareness of the risk factors [18].

Low education levels and substance use/abuse, including cigarette smoking, betel chewing, and alcohol consumption, have been identified as significant risk factors for esophageal cancer in Taiwan [40]; betel nut chewing is the main independent risk factor. The more betel nuts eaten in a lifetime, the higher the cancer risk. According to a previous study, people who both smoke cigarettes and drink alcohol excessively are 20.4-times more likely to have esophageal cancer than people who do not have these habits. Moreover, people who have the combined habits of betel chewing, cigarette smoking, and alcohol consumption have a 41.2-times higher risk than those who do not have any of these habits [40]. Therefore, betel chewing was a key factor and was significantly associated with ESCC incidence rates. The prevalence of betel chewing in Taiwan exceeds 10% [41], predominantly among men, which probably at least partially explains the dramatic difference in the incidence rate of esophageal cancer between men and women. Taiwan’s healthcare system, the NHI, is a mandatory, single-payer insurance system. Patients in Taiwan have complete freedom of choice among providers when they seek care and benefit from low deductible expenses. Patients rarely suffer from long wait times for health care services, and their access to care is protected by multiple measures. Almost all hospitals have joined the system to serve this highly competitive and efficient medical market [22], and NHI fully covers the necessary diagnostic tests and routine treatments. In Taiwan, routine cancer screening programs include fecal occult blood test for colorectal cancer, oral mucosal screen for oral cancer, Pap smear for cervical cancer and mammography for breast cancer. Esophageal cancer is the sixth leading cause of cancer-related death in Taiwan and upper gastrointestinal endoscopy (UGIE) is the gold diagnostic standard. Based on cost-effectiveness, the government cannot provide large-scale screening for early diagnosis [42]. However, esophageal cancer runs an insidious course before symptoms appear, and prognosis is adversely affected by late-stage diagnosis. Early-stage esophageal cancer tends to be asymptomatic; dysphagia occurs only when the lumen of the esophagus becomes constricted to less than 14 mm, thus, symptomatic cases are generally diagnosed in late-stage. The more favorable tumor staging in high SES population is presumably benefitted from routine self-pay physical checkups which regularly cover UGIE in the package. In this study, the incidence of esophageal cancer was significantly lower in the highest income group; perhaps periodic endoscopic screening through self-pay routine physical checkups is responsible for this result. Unfortunately, despite the numerous studies conducted throughout the world, no non-endoscopic screening for esophageal cancer has been found to be practical and effective. Screening endoscopy that is focused on the most at-risk populations may be a reasonable alternative.

### 4.1. Weaknesses and Limitations

We acknowledge that this study has weaknesses and limitations. First, 25% of the targeted population (Figure 1), which belongs to the other 12 of the 15 social-status categories, was excluded because of disparities in lifestyle and socioeconomic conditions, which could have led lead to bias in the data analysis. Second, in the NHIRD, salary data are divided into 54 levels, and each level contains a different number of people. The distribution of income among all beneficiaries is highly positively skewed, which makes affirming significant income steps and attaining an even case number in each group for comparison impossible. Therefore, income is sorted mainly into three groups, representing relatively high (24.2%), medium (20.6%), and low income (55.2%) levels, and this might also cause bias. Third, the legislated age for retiring is 65 years old in Taiwan, but our study covers only subjects between 40 and 79 years old, so the SES cannot be reflective of age above 65, either in income or occupation. Fourth, although using the education index to perform SES-related studies is a common practice, such information is not recorded in the NHIRD; therefore, we had to search for an alternative.

Unfortunately, there are some methodological limitations of this study. The main risk factors for esophageal cancer such as obesity, chronic gastroesophageal reflux disease (GERD) and Barrett’s esophagus were could not be recorded from the NHIRD. Additionally, the identified risk factors for esophageal cancer, such as smoking, alcohol consumption and betel chewing, were not accurately recorded, so the exact power of the linkage could not be verified. In our future study, we hope to collect the health-related behaviors of individuals from the national database on cancer screening to provide a reference for a cost-effective screening program. This study only discloses one of many aspects; to provide a solid foundation for sound public health strategies, it is crucial to conduct more studies from other health outcome indicators, e.g., choice of therapeutic modalities, prognosis, and mortality.

### 4.2. Strengths and Contributions

The extreme geographical and temporal histological variations in the incidence rates of esophageal cancer require that approaches to the disease conform to the demographic base. The NHIRD is an integrated system that provides comprehensive healthcare data for analysis. In this nationwide large-scale retrospective study, we applied two SES indexes and two histologic types, which provided us the opportunity to assess the individual and comprehensive effects of various outcome indicators [12]. To our knowledge, this is the first Taiwanese study to examine the role of socioeconomic disparities in the incidence and late-stage diagnosis of esophageal cancer. Socioeconomic inequalities in cancer mortality are considered meaningful for malignancies that are amenable to prevention or treatment. With the high medical accessibility in Taiwan, the treatment of esophageal cancer after diagnosis is likely equitable. Attempts to minimize the impact of the disease should be focused on prevention and early diagnosis, such as cessation of smoking, alcohol drinking, and betel chewing; healthy living; and cancer screening programs, such as upper gastrointestinal endoscopy, especially targeted at disadvantaged groups. Additionally, this study highlights the need for updated data on differences in esophageal cancer between Asian and Western countries.

## 5. Conclusions

A nationwide, large-scale retrospective analysis, which retrieved nearly 8000 eligible esophageal cancer cases from 2008 to 2012 in Taiwan, was conducted. Major features of esophageal cancer in the country were identified: increase in recent decades; high preponderance of ESCC over EAC in histological type; extreme male predominance; peak incidence of ESCC between 50–59 years; and a trend toward increasing incidence of EAC with age. This is the first Taiwanese study to demonstrate the role of socioeconomic disparities in the incidence and late-stage diagnosis of esophageal cancer. Low-SES populations have a disproportionately higher incidence and a higher percentage of late-stage diagnosis. Despite high medical accessibility in Taiwan, SES disparities still lead to significant disadvantages in the incidence and late diagnosis of esophageal cancer. The increasing socioeconomic disparities in Taiwan and narrowing the gaps in health in equities are issues that must be addressed.

## Figures and Tables

**Table 1:** Characteristics of patients with esophagus cancer (*n* = 7763).

Variables	Total (*n* = 7763)		Squamous Cell Carcinoma (*n* = 7500)		Adenocarcinoma (*n* = 263)	
	Number	Percent (%)	Number	Percent (%)	Number	Percent (%)
Age, years						
40–49	2458	(31.7)	2409	(32.1)	49	(18.6)
50–59	2974	(38.3)	2889	(38.5)	85	(32.3)
60–69	1503	(19.4)	1439	(19.2)	64	(24.3)
70–79	828	(10.7)	763	(10.2)	65	(24.7)
Gender						
Female	431	(5.6)	394	(5.3)	37	(14.1)
Male	7332	(94.4)	7106	(94.7)	226	(85.9)
Occupation						
Labor (Industry)	5004	(64.5)	4850	(64.7)	154	(58.6)
Enterprise employee (Private and business)	2475	(31.9)	2381	(31.7)	94	(35.7)
Government employee (Public)	284	(3.7)	269	(3.6)	15	(5.7)
Income, TWD						
17,281–21,000	4289	(55.2)	4154	(55.4)	135	(51.3)
21,001–31,800	1598	(20.6)	1555	(20.7)	43	(16.3)
>31,800	1876	(24.2)	1791	(23.9)	85	(32.3)

Values are expressed as numbers (percentages).

**Table 2:** Incidence rate (per 100,000 person-years) of esophagus cancer in Taiwan, 2008–2014.

	Squamous Cell Carcinoma					Adenocarcinoma				
	Event	PY	Incident Rate	95% CI		Event	PY	Incident Rate	95% CI	
Occupation										
Labor (Industry)	4850	244,39,015	19.85	19.29	20.41	154	24,421,822	0.63	0.53	0.74
Enterprise employee (Private and business)	2381	19,622,489	12.13	11.65	12.63	94	19,614,037	0.48	0.39	0.59
Government employee (Public)	269	3,508,761	7.67	6.78	8.64	15	3,507,855	0.43	0.24	0.71
Income, TWD										
17,281–21,000	4154	20,195,912	20.57	19.95	21.20	135	20,181,243	0.67	0.56	0.79
21,001–31,800	1555	10,043,646	15.48	14.72	16.27	43	10,037,993	0.43	0.31	0.58
>31,800	1791	17,330,708	10.33	9.86	10.82	85	17,324,478	0.49	0.39	0.61
Total	7500	47,570,266	15.77	15.41	16.13	263	47,543,714	0.55	0.49	0.62

CI, confidence interval.

**Table 3:** Distribution of stage in patients with esophagus cancer (*n* = 6672).

Variables	Squamous Cell Carcinoma (*n* = 6453)		Adenocarcinoma (*n* = 219)	
	Number	Percent (%)	Number	Percent (%)
Stage				
0	58	(0.9)	0	(0.0)
1	344	(5.3)	22	(10.0)
2	1062	(16.5)	39	(17.8)
3	3132	(48.5)	71	(32.4)
4	1857	(28.8)	87	(39.7)

Values are expressed as numbers (percentages).

**Table 4:** Univariate and multivariable Cox proportional hazard models of esophagus cancer incidence.

Variables	Squamous Cell Carcinoma						Adenocarcinoma					
	Univariate Models			Full Model			Univariate Models			Full Model		
	HR	95% CI	*p*-Value	HR	95% CI	*p*-Value	HR	95% CI	*p*-Value	HR	95% CI	*p*-Value
Age, years												
40–49	1.00	-	-	1.00	-	-	1.00	-	-	1.00	-	-
50–59	1.52	(1.44–1.61)	<0.001	1.47	(1.39–1.55)	<0.001	2.20	(1.55–3.13)	<0.001	2.18	(1.53–3.1)	<0.001
60–69	1.66	(1.55–1.77)	<0.001	1.44	(1.35–1.54)	<0.001	3.62	(2.5–5.26)	<0.001	3.50	(2.4–5.1)	<0.001
70–79	1.33	(1.23–1.45)	<0.001	1.18	(1.08–1.28)	<0.001	5.60	(3.86–8.11)	<0.001	5.69	(3.89–8.33)	<0.001
Gender												
female	1.00	-	-	1.00	-	-	1.00	-	-	1.00	-	-
male	18.12	(16.37–20.05)	<0.001	19.25	(17.39–21.3)	<0.001	6.15	(4.34–8.7)	<0.001	6.37	(4.5–9.03)	<0.001
Occupation												
Labor (Industry)	1.00	-	-	1.00	-	-	1.00	-	-	1.00	-	-
Enterprise employee (Private and business)	0.61	(0.58–0.64)	<0.001	0.76	(0.72–0.81)	<0.001	0.76	(0.59–0.98)	0.035	0.91	(0.64–1.31)	0.626
Government employee (Public)	0.39	(0.34–0.44)	<0.001	0.58	(0.51–0.67)	<0.001	0.68	(0.4–1.15)	0.150	0.78	(0.42–1.45)	0.429
Income, TWD												
17,281–21,000	1.00	-	-	1.00	-	-	1.00	-	-	1.00	-	-
21,001–31,800	0.75	(0.71–0.8)	<0.001	0.91	(0.86–0.97)	0.005	0.64	(0.45–0.9)	0.011	0.93	(0.63–1.36)	0.694
>31,800	0.50	(0.48–0.53)	<0.001	0.56	(0.52–0.6)	<0.001	0.73	(0.56–0.96)	0.024	0.92	(0.62–1.39)	0.706

HR, hazard ratio; CI, confidence interval.

**Table 5:** Univariate and multivariable logistic regression models of late-stage (Stage III + IV) diagnosis in patients with esophagus cancer.

Variables	Squamous Cell Carcinoma						Adenocarcinoma					
	Univariate Models			Full Model			Univariate Models			Full Model		
	OR	95% CI	*p*-Value	OR	95% CI	*p*-Value	OR	95% CI	*p*-Value	OR	95% CI	*p*-Value
Age, yrs												
40–49	1.00	-	-	1.00	-	-	1.00	-	-	1.00	-	-
50–59	1.05	(0.91–1.2)	0.539	1.07	(0.93–1.24)	0.327	1.52	(0.65–3.54)	0.335	1.65	(0.69–3.95)	0.259
60–69	0.88	(0.75–1.04)	0.142	0.90	(0.76–1.06)	0.197	2.65	(0.99–7.09)	0.053	2.68	(0.98–7.33)	0.055
70–79	0.80	(0.66–0.98)	0.032	0.86	(0.7–1.05)	0.143	0.97	(0.4–2.33)	0.945	0.81	(0.32–2.04)	0.653
Gender												
female	1.00	-	-	1.00	-	-	1.00	-	-	1.00	-	-
male	1.94	(1.53–2.45)	<0.001	1.88	(1.48–2.38)	<0.001	0.48	(0.17–1.3)	0.148	0.47	(0.16–1.35)	0.159
Occupation												
Labor (Industry)	1.00	-	-	1.00	-	-	1.00	-	-	1.00	-	-
Enterprise employee (Private and business)	0.97	(0.86–1.1)	0.659	1.07	(0.91–1.26)	0.395	0.98	(0.53–1.82)	0.954	1.61	(0.67–3.86)	0.291
Government employee (Public)	0.97	(0.71–1.33)	0.851	1.19	(0.84–1.68)	0.335	0.63	(0.14–2.76)	0.538	1.29	(0.22–7.39)	0.775
Income, TWD												
17,281–21,000	1.00	-	-	1.00	-	-	1.00	-	-	1.00	-	-
21,001–31,800	0.99	(0.85–1.15)	0.904	0.94	(0.8–1.1)	0.443	0.84	(0.36–1.95)	0.679	0.57	(0.22–1.49)	0.252
>31,800	0.88	(0.77–1.02)	0.086	0.82	(0.68–0.98)	0.034	0.63	(0.33–1.22)	0.169	0.39	(0.14–1.03)	0.057

OR, odds ratio; CI, confidence interval.

## Data Availability

The detailed information on the program and data access is available from the NHIRD and TCR are not publicly available. The authors confirm that, for approved reasons, some access restrictions may apply to the data underlying the findings. The data used in this study cannot be made available in the manuscript, the supplemental files, or in a public repository due to the Personal Information Protection Act executed by Taiwan’s government, starting in 2012. Requests for data can be sent as a formal proposal to obtain approval from the ethics review committee of the appropriate governmental department in Taiwan.
